# Associated factors of repeated suicidal behavior

**DOI:** 10.1192/j.eurpsy.2022.2188

**Published:** 2022-09-01

**Authors:** O. Charaa, A. Aissa, N. Sayari, Z. Yosra, S. Meddouri, U. Ouali, R. Jomli

**Affiliations:** 1 Razi hospital, Psychiatry A Department, manouba, Tunisia; 2 Razi hospital, Psychiatry A Department, Manouba, Tunisia

**Keywords:** Associated factors, Suicide, Recidivism

## Abstract

**Introduction:**

Suicide is a dramatic suicidality complication and a significant worldwild public health problem. Sixty percent of suicidal deaths are preceded by at least one suicide attempt.

**Objectives:**

to search and estimate the factors predicting a suicidal recidivism

**Methods:**

We conducted a retrospective descriptive survey, achieved in psychiatric departement A of Razi hospital on 60 patients hospitalized during a period of 10 years (from January 2010 to December 2019) and have committed at least a suicide attempt. Data collected from medical folders in order to explore sociodemographic and clinical characteristics of the patients.

**Results:**

The mean age of the sample was 30 years. A high prevalence of female was objectified. There were a low level of education for 53%, most of patients (55%) were unemployed and came from urban area. Among our patients, 39% attempted suicide for a one time. 61% of patients attempted suicide for several times. The main risk factors related to recidivism of suicidal behavior were unemployment, family history of psychiatric disorders and family instability.

**Conclusions:**

The analysis of these results justifies preventive actions in order to face the increase of suicidal recidivism by searching for these associated factors. Therefore, a multidisciplinary intervention approach is required.

**Disclosure:**

No significant relationships.

